# Malnutrition upon Hospital Admission in Geriatric Patients: Why Assess It?

**DOI:** 10.3389/fnut.2017.00050

**Published:** 2017-10-30

**Authors:** Paolo Orlandoni, Claudia Venturini, Nikolina Jukic Peladic, Annarita Costantini, Mirko Di Rosa, Claudia Cola, Natascia Giorgini, Redenta Basile, Donata Fagnani, Debora Sparvoli, Serenella David

**Affiliations:** ^1^Clinical Nutrition, Istituto Nazionale di Riposo e Cura per Anziani (IRCCS), Ancona, Italy

**Keywords:** geriatrics, in-hospital patients, nutritional screening, malnutrition, hospitalization outcomes

## Abstract

**Objective:**

To assess the prevalence of malnutrition according to the new ESPEN definition in a population of geriatric hospital patients and to determine how malnutrition affects the length of hospital stay (LOS) and hospital mortality.

**Design:**

A retrospective analysis of data gathered during nutritional screening surveys carried out three consecutive years, from 2012 to 2014, in an Italian geriatric research hospital (INRCA, Ancona) was performed. On the day of the study, demographic data, data on clinical conditions and the nutritional status of newly admitted patients were collected. Patients were screened for malnutrition risk using the Malnutrition Universal Screening Tool (MUST). Subsequently, malnutrition was diagnosed, for subjects at high risk, following the criteria suggested by the European Association for Clinical Nutrition and Metabolism [body mass index (BMI) < 18.5 kg/m^2^ or different combinations of unintentional weight loss over time and BMI values]. Sensitivity, specificity, positive and negative predictive value of MUST compared to ESPEN criteria were assessed. The characteristics of patients with a diagnosis of malnutrition were compared to those of non-malnourished patients. The impact of malnutrition on LOS and hospital mortality was investigated through logistic and linear regression models.

**Setting:**

The study was performed in an Italian geriatric research hospital (INRCA, Ancona).

**Subjects:**

Two hundred eighty-four newly hospitalized geriatric patients from acute care wards (mean age 82.8 ± 8.7 years), who gave their written consent to participate in the study, were enrolled.

**Results:**

According to the MUST, high risk of malnutrition at hospitalization was found in 28.2% of patients. Malnutrition was diagnosed in 24.6% of subjects. The malnutrition was an independent predictor of both the LOS and hospital mortality. The multivariate analyses—linear and logistic regression—were performed considering different potential confounders contemporarily. The results showed that the malnutrition is an independent predictor of LOS and hospital mortality. Malnourished subjects were hospitalized almost 3 days longer compared to non-malnourished patients (*p* = 0.047; CI 0.04–5.80). The risk of death during hospitalization was 55% higher for malnourished patients (*p* = 0.037; CI 0.21–0.95).

**Conclusion:**

A new ESPEN consensus of malnutrition was easily applicable in a population of geriatric hospital patients. Given that the nutritional status of geriatric patients was strongly correlated with the LOS and hospital mortality, the use of this simple and non-time consuming tool is highly recommended in clinical practice.

## Introduction

Malnutrition is a broad term used to define different deviations from a normal nutritional state. The imbalance between nutritional intake and nutritional requirements may result in undernutrition (deficiency of one or more essential nutrients) or in overnutrition (excess of nutrients) (1–3). Protein-energy undernutrition, in particular, is a state of energy and protein insufficiency resulting either from reduced nutrient intake, impaired absorption and assimilation, increased energy expenditure, or a combination of these (4, 5). There is an extensive scientific literature on the clinical and economic consequences of protein-energy malnutrition (6, 7). It has been determined that malnutrition compromises the immune response and increases the risk of infections and infection-related complications, slows down the wound-healing process, delays recovery from illness, prolongs hospitalization, and increases the risk of death (8–10). Once the correlation between malnutrition and clinical outcomes was discovered, nutritional screening of hospital patients became mandatory in numerous countries. In order to develop an appropriate nutritional plan and intervene immediately, the screening of patients’ nutritional status is normally performed on hospital admission. For this purpose, numerous screening tools have been developed and validated (11, 12). Some studies found that more than 90% of newly admitted geriatric patients present with protein-energy malnutrition or are at risk of malnutrition (13, 14). During hospitalization, numerous conditions—underlying diseases, comorbidity, inflammatory states, and infections—increase patients’ energy expenditure, while reducing their normal intake (15–17). Therefore, during the later stages of hospitalization all patients should undergo an assessment of their nutritional status. The lack of an internationally accepted criterion for the diagnosis of malnutrition and the use of different indicators, some of which have been rejected by the most recent scientific evidence, probably explain a wide range of malnutrition prevalence rates reported in the literature. These rates vary from 20 to 60% in acute care among geriatric patients (18–20). In Italian hospitals, unfortunately, relatively little attention is given to nutritional issues, and neither nutritional screening, nor assessment, are routinely performed at hospital admission. Consequently, there are no reliable data on the nutritional status of in-hospital Italian patients, which is one of the oldest population worldwide. This manuscript presents the results of a retrospective analysis of data gathered during 1-day nutritional screening surveys. The surveys were performed for three consecutive years (2012–2014) in a geriatric research hospital—INRCA, Ancona–with the aim of assessing the prevalence of malnutrition risk among geriatric patients using the Malnutrition Universal Screening Tool (MUST). The data were also analyzed in light of the recently released consensus definition of malnutrition of the European Association for Clinical Nutrition and Metabolism (ESPEN) to assess the prevalence rate of malnutrition (21). To detect factors associated with malnutrition, the baseline characteristics of malnourished patients were confronted with those of patients without a malnutrition diagnosis. Multivariate analyses were performed to assess the impact of malnutrition on the length of hospital stay (LOS) and hospital mortality. The term malnutrition was exclusively used, synonymously, with undernutrition.

## Materials and Methods

### Settings and Sample

The Clinical Nutrition Unit, and the Medical Directorate, of the geriatric hospital INRCA-Ancona promoted a 1-day cross-sectional study among INRCA patients, for three consecutive years (2012–2014). After obtaining ethics committee approval, all newly admitted patients—i.e., patients admitted in the previous 48 h—were invited to participate in the study. Patients hospitalized in the emergency room and patients in post-acute care (long-term care and rehabilitation) were excluded. All patients gave their written informed consent for the collection and processing of their data for scientific purposes. Data on the outcomes of hospitalization were extrapolated from hospital archives.

### Data Collection

A specific questionnaire was developed to collect demographic data (living arrangements, birth date, gender), data on patients’ clinical conditions (pathologies, disorders and comorbidities, immobilization, pressure ulcers, dysphagia, edentulism, history of weight loss, information on previous hospitalizations) and data on nutrition prescribed and administered during a hospitalization (diets and feeding routes). All questionnaires were filled out by trained staff (physicians, dieticians, and nurses) with the help of caregivers for non-collaborative patients. In addition, patients’ anthropometric data were gathered. Body weight was measured to the nearest 0.1 kg and height was determined to the nearest 0.1 cm, in subjects wearing light pajamas and without shoes. Height was measured with a height rod. Bedridden patients were weighed by bed scale and a special weighing set, complete with a digital scale and support spreader bar (Help 2000, Tassinari balance Srl). Bedridden patients’ heights were estimated from ulna length according to tables provided in the appendix of the MUST screening tool (22). The reduction of muscle mass was assessed from calf circumference, evaluated with a flexible tape measure at the proximal base of the inferior limb, under the knee, at the point of greatest diameter. On the same day, patient blood samples were collected by nurses and measurements of serum albumin (Alb) and serum prealbumin (Pab) were carried out in the hospital blood analysis laboratory. Patients’ body mass index (BMI) and Geriatric Nutrition Risk Index (GNRI), a tool to predict the risk of morbidity and mortality in hospitalized older patients, were determined based on collected data (23). Dysphagia screening, on admission, was performed by each ward with a bedside assessment technique. Data on the outcomes of hospitalization were gathered from hospital archives.

### Malnutrition Screening

Malnutrition screening was performed with MUST. This tool allows the identification of patients with different levels of malnutrition risk (low, moderate, and high) and includes a management plan for nutritional intervention. The first step involves assigning a score to the patient’s BMI. The second step consists of setting up the patient’s unplanned Weight loss Score. During the third step, the acute disease effect score is assigned to each patient. The fourth step involves the estimate of malnutrition risk, which is calculated by the sum of single scores (0 = no risk, 1 = medium risk; score ≥ 2 = high risk). For each score, the management plan is provided within the fifth step. MUST is a validated and internationally accepted screening tool, which is easy to use and quick to perform. Its compilation requires from 3 to 5 min. It is particularly suited for hospital use both for its simplicity and because the effect of acute disease is considered.

### Malnutrition Diagnosis

According to the recent consensus definition released by ESPEN, the diagnosis of malnutrition is a two-step process. After fulfilling the criteria for being at risk of malnutrition, by any validated risk screening tool, which is mandatory, those who are identified, proceed in the diagnostic process. The diagnosis may be performed in two optional ways. The first option requires a BMI < 18.5 kg/m^2^. The second option encompasses unintentional weight loss (UwL) (>10% independent of time or >5% in the last 3 months), always combined with either a low BMI (<20 kg/m^2^ if <70 years old or <22 kg/m^2^ if ≥70 years old) or a low Fat Free Mass index (FFMI; <15 kg/m^2^ for women and <17 kg/m^2^ for men). In this study, malnutrition was diagnosed for patients who were at high risk of malnutrition according to the MUST (score ≥ 2) if they fulfilled one of the following criteria: BMI < 18.5 kg/m^2^ and UwL > 10% undefined length of time or >5% over the last 3 months, combined with BMI < 22 kg/m^2^. Data on patients’ FFMI were not available. Sensitivity and specificity of MUST tool compared to ESPEN definition for diagnosis of malnutrition were calculated to detect the ability of the MUST tool to detect the malnutrition (sensitivity) and its ability not to give a positive result when patients are not malnourished. Positive and negative predictive values were calculated to assess how many of the subjects whose test is positive truly are malnourished and how many of the subjects whose test was negative actually were not malnourished.

### Statistical Analyses

Descriptive statistics were used to describe patient characteristics. Continuous variables were expressed as mean values ± SD. Categorical variables were expressed as relative frequencies. Chi-square, odds ratio, and *t*-test were used to describe differences between malnourished and non-malnourished patients. Kolmogorov–Smirnov test was used to verify if the continuous variables included in the multivariate analyses had a normal distribution. The impact of malnutrition on LOS was investigated using the linear regression model with LOS (measured as number of days spent in the hospital) as the dependent variable and risk of malnutrition (according to the ESPEN definition) as the independent variable. The correlation between malnutrition and the outcome variable LOS was adjusted for gender, age, and comorbidities. The impact of malnutrition on the outcomes of hospitalization—hospital mortality—was investigated through the logistic regression model with the outcome of hospitalization (death or discharge from hospital) as the dependent variable and risk of malnutrition as independent variable. The correlation between the malnutrition and the outcome variable “in hospital mortality” was adjusted for different pathologies (diagnoses). Statistical significance of estimated coefficients was assessed through the *t*-test. The validity and overall reliability of the model was evaluated by the *F*-test and *R*-squared. The level of significance for all tests was set at *p* < 0.05. Data collected were analyzed with the Statistical Package for Social Sciences (SPSS) version 19.0.

## Results

The analyses included 284 patients, of which 51.0% were females and 49.0% were males. The patients had a mean age of 82.8 ± 8.7; 50% of the subjects were ≥85 years, the so-called “the oldest old.” Following malnutrition risk screening, performed with MUST, 28.2% of patients had a high risk of malnutrition. For 88.3% of those patients at high risk, or 24.6% of the total population, malnutrition was diagnosed using the new ESPEN consensus definition (Table 1). We assessed that MUST has a sensitivity of 98%, specificity of 96%, positive predictive value of 89%, and negative predictive value of 99% compared to ESPEN definition.

**Table 1:** Prevalence of high risk of malnutrition [Malnutrition Universal Screening Tool (MUST)] and of malnutrition (ESPEN consensus definition on malnutrition).

	Absolute frequencies	Relative frequencies
Total sample	284	100%
Screened at high risk of malnutrition according to MUST (≥2)	80	28.2%
Malnourished according to ESPEN consensus definition	70	24.6%

The relevance of nutritional problems was confirmed by the mode of feeding and the types of hospital diet registered on the study days. Almost 13% of patients were artificially fed (by Nasogastric tube, Percutaneous endoscopic gastrostomy and Venous catheter), mostly because of severe dysphagia. Texture-modified diets were prescribed for 24.4% of patients and pathology-specific diets were prescribed for 22.6%. More than 7% of patients were prescribed light, pre- and post-operative diets, or were fasting prior to medical tests and blood analyses. Only 33.0% of patients consumed a standard 2,000 kcal diet comprising 18% proteins, 28% fat, and 54% carbohydrates.

To identify factors associated with malnutrition, the baseline characteristics of malnourished and non-malnourished patients were compared (Table 2).

**Table 2:** Descriptive analysis of baseline sociodemographic, functional, and clinical parameters in the whole sample and in malnourished and non-malnourished patients—relative frequencies (%) and mean values (±SD).

	Total sample (*n* = 284)	Malnourished patients (*n* = 70)	Well-nourished patients (*n* = 214)	*p*
	Relative frequencies (%)/mean values	Relative frequencies (%)/mean values	Relative frequencies (%)/mean values	
Age (mean ± SD)^a^	82.8 ± 8.7	85.6 ± 7.4	81.9 ± 9.0	0.0027
Gender^b^	F 48.8%; M 51.2%	51.5% F; 48.5% M	52.1% F; 47.9% M	0.6160
Setting^b^	88.0% Home; 8.5% N. Home; 3.5% Other hospital	86.6% Home; 10.4% N. Home; 3.0% Other hospital	88.5% Home; 7.8% N. Home; 3.7% Other hospital	0.7830
Pressure ulcers^b^	23.3% Yes; 76.7% No	37.9% Yes; 62.1% No	18.6% Yes; 81.4% No	0.0037
Bedridden^b^	43.0% Yes; 57.0% No	61.2% Yes; 38.8% No	36.7% Yes; 63.3% No	0.0000
Dysphagia^b^	45.3% Yes; 54.7% No	54.9% Yes; 45.1% No	41.3% Yes; 58.7% No	0.0720
Edentulism^b^	47.8% Yes; 52.2% No	45.6% Yes; 54.4% No	48.7% Yes; 51.3% No	0.6600
Calf circumference (mean ± SD)^a^	31.6 ± 4.7	27.9 ± 4.1	33.0 ± 4.1	0.0000
Dementia^b^	13.7% Yes; 86.3% No	19.1% Yes; 80.9% No	11.9% Yes; 88.1% No	0.1340
Chronic obstructive pulmonary disease^b^	5.3% Yes; 94.7% No	5.9% Yes; 94.1% No	5.1% Yes; 94.9% No	0.8180
Diabetes mellitus type 2^b^	12.6% Yes; 87.4% No	8.8% Yes; 91.2% No	13.9% Yes; 86.1% No	0.2760
Respiratory insufficiency^b^	0.4% Yes; 99.6% No	0.0% Yes; 100.0% No	0.5% Yes; 99.5% No	0.5530
Chronic kidney disease^b^	17.6% Yes; 82.4% No	20.6% Yes; 79.4% No	16.5% Yes; 83.5% No	0.4450
Myocardial infarct^b^	1.5% Yes; 98.5% No	0.0% Yes; 100.0% No	2.0% Yes; 98.0% No	0.2330
Cancer^b^	9.9% Yes; 90.1% No	7.4% Yes; 92.6% No	10.8% Yes; 89.2% No	0.4100
Other neurological diseases^b^	26.0% Yes; 74.0% No	27.9% Yes; 72.1% No	25.3% Yes; 74.7% No	0.6640
Heart failure^b^	26.7% Yes; 73.3% No	23.5% Yes; 76.5% No	27.8% Yes; 72.2% No	0.4900
Comorbidities	37.9% Yes; 62.1% No	41.8% Yes; 58.2% No	36.6% Yes; 63.4% No	0.4500
Body mass index (mean ± SD)^a^	24.0 ± 4.9	19.5 ± 3.9	25.6 ± 4.1	0.0000
Unintentional loss of weight^b^	38.6% Yes; 61.4% No	94.9% Yes; 5.1% No	20.3% Yes; 79.7% No	0.0000
Albumin g/ml (mean ± SD)^a^	3.0 ± 0.7	2.7 ± 0.7	3.1 ± 0.7	0.0000
Pre albumin mg/ml (mean ± SD)^a^	13.3 ± 6.6	10.5 ± 6.1	14.3 ± 6.5	0.0001
Geriatric Nutrition Risk Index (mean ± SD)^a^	90.2 ± 11.4	81.6 ± 11.9	93.6 ± 9.4	0.0000
Hospitalizations in previous 6 months^b^	43.1% Yes; 56.9% No	61.5% Yes; 38.5% No	36.8% Yes; 63.2% No	0.0070
Duration of hospitalization (mean ± SD)^a^	13.1 ± 10.0	16.0 ± 12.5	12.1 ± 8.8	0.0085
Death during hospitalization^b^	16.3% Yes; 83.7% No	24.2% Yes; 75.8% No	13.5% Yes; 86.5% No	0.0490

*^a^t-test*.

*^b^Chi squared test*.

Malnourished patients more frequently had symptoms of oropharingeal dysphagia, were older and bedridden, or were hospitalized in the previous 6 months. Statistically significant differences were also found between the two groups in the mean values of all the single indicators of nutritional status; GNRI, BMI, UwL, and between Alb and Pab values. The mean LOS for the sample population was 13.1 ± 10.0 days, while the overall hospital mortality rate was 16.3% with important differences in outcomes between the two groups of patients. To estimate the impact of the variable “malnutrition,” on the outcome variable “hospital mortality,” a multiple logistic regression analysis was performed (see Table 3). The results showed that the risk of dying during hospitalization was 55% higher for malnourished patients compared to non-malnourished subjects (*p* = 0.037; CI 0.21–0.95). The risk of hospital mortality was also strongly correlated with a cancer diagnosis; patients with cancer had a 68% higher probability of dying during hospitalization compared to other patients (*p* = 0.036; CI 0.11–0.93).

**Table 3:** Impact of malnutrition diagnosed using the ESPEN consensus definition on the outcomes of hospitalization.

	Coefficient	95% Confidence interval
Malnutrition*	2.92	0.04–5.80
Female gender	−2.19	−4.66–0.29
Age**	0.22	0.08–0.36
Comorbidities	1.50	−1.06–4.05
Constant	−5.19	−16.67–6.30

*0.010 < p < 0.050.

**p < 0.001.

*N = 255, R-squared = 0.077*.

The results of the linear regression analysis also showed that malnutrition was statistically significantly correlated with the LOS (Table 4). In fact, malnourished subjects, on average, spent almost 3 more days in the hospital compared with non-malnourished patients (*p* = 0.047; CI 0.04–5.80). Among control variables, only age was significantly correlated with the dependent variable; when age increased approximately 5 years, the LOS increased by 1 day. Gender and comorbidities were not significantly associated with the dependent variable (LOS).

**Table 4:** Impact of malnutrition diagnosed using ESPEN consensus definition on the length of hospital stay.

	Odds ratio	95% Confidence interval
Malnutrition*	0.45	0.21–0.95
Diagnoses		
Dementia	1.64	0.50–5.39
Myocardial infarction	1.20	0.49–2.95
Cancer*	0.32	0.11–0.93
Renal insufficiency	0.77	0.28–2.08
Neurological disorders	0.73	0.31–1.72
Diabetes	5.86	0.72–47.36
Infection	0.40	0.14–1.19

*0.010 < p < 0.050.

*N = 255, R-squared = 0.063*.

## Discussion

Even though it has been repeatedly determined that protein-energy malnutrition can compromise clinical outcomes of hospitalized geriatric patients, screening and assessment of the nutritional status of this patient population is not routinely carried out in Italian hospitals (24, 25). Consequently, no reliable data on the prevalence of malnutrition among geriatric patients are available (26, 27). This important gap in knowledge stimulated the Clinical Nutrition Unit of the geriatric hospital, INRCA of Ancona, to carry out a 1-day nutritional screening survey, among newly admitted patients, for three subsequent years (2012–2014). The primary aim of the surveys was to collect reliable data on malnutrition risk at hospital admission. The MUST screening tool was used in each of the surveys (28, 29). Subsequently, a diagnosis of malnutrition was performed, for all patients at high risk, following the latest ESPEN consensus statement on the definition of malnutrition. The screening identified 28.2% of patients at high risk of malnutrition, of whom 88.3% suffered from malnutrition (24.6% of the total population). Unfortunately, the comparison of our results on malnutrition prevalence, with the results of other published studies, is very difficult due to the use of different screening and assessment tools and the specific populations analyzed. In some studies, significantly higher rates of malnutrition risk were found [Persson et al. (13), Thomas et al. (14)]. Other studies found rates that were quite similar to those reported in our investigation [Imoberdorf et al. (19), Sheen et al. (30)]. Our results showed a strong correlation between malnutrition and hospital mortality, with a 68% higher probability of malnourished patients dying during hospitalization. Previous evidence on this relation are contrasting. Numerous studies have identified malnutrition as an important predictive factor of in hospital mortality [Sullivan et al. (15), Caccialanza et al. (31), Gallagher-Allred et al. (32)], while other investigations find that the malnutrition upon hospitalizations is a predictive factor of mortality after hospital discharge [Lim et al. (7), Liu et al. (9), Personn et al. (13)]. According to our results, malnutrition at hospital admission also strongly influenced the LOS, which, on average, was 3 days longer for patients who were malnourished. This result coincides with the results of previous studies (Lim et al., Cederholm et al.) (7, 33). The present study provides also the first evidence of applying the novel ESPEN consensus definition of malnutrition in a population of hospitalized geriatric patients. The ESPEN approach was easily applicable. Only the measurement of FFM may represent some problem. It is so because, in some patients who are not collaborative and may have important physical impairments, it may be quite difficult to gather a reliable data. In addition, it has to be mentioned that BIA is not always available. The lack of data on FFM could result in the exclusion of an important portion of sarcopenic patients who have a normal BMI and no weight loss, but who have low FFM. According to the ESPEN consensus definition, any validated screening tool may be used to identify patients at risk of malnutrition. Different screening tools will vary in classifying patients according to malnutrition risk. The MUST tool, for example, defines three categories of risk. In this study, the second step of screening was performed only for patients at high risk of malnutrition (MUST score ≥ 2). Considering only the high risk group of patients, we found that MUST tool has an excellent sensitivity and specificity as well as positive and negative predictive values compared to ESPEN definition. It would be important to clarify, for each screening tool, the categories of patients for which a malnutrition diagnosis should be performed.

Some study limitations have to be mentioned. First, this is a retrospective study and, as it is known, retrospective studies have more potential sources of bias and confounding than the prospective ones. The investigation concentrated on malnutrition at hospital admission. But, it is well known that malnutrition frequently occurs during hospitalization, which may involve patients who are not at risk on admission. Considering that our study was performed in hospitalized patients, hospital-acquired malnutrition should have also been investigated. In addition, patients’ food intake should have been assessed. To overcome the latter limitation, hospital menus and patients’ food intake were investigated and confronted with their requirements during the last survey in 2014, but given the limited number of observations collected, the results of that analysis were not presented. This investigation was performed with relatively low number of participants; future studies on larger populations should be carried out to confirm our findings. No information on functional independence of patients were collected in our study but that information should be gathered in order to test how it influences the LOS and hospital mortality. Considering that the inflammation is also an important etiologic factor for malnutrition, data on inflammatory markers would also be useful and their correlation to LOS and hospital mortality should be tested. The readmission rates and post-discharge mortality (34) as well as the costs and benefits of nutritional interventions should also be evaluated in the future (35).

## Ethics Statement

The study was approved by Ethics Commitee of INRCA IRCCS (Ancona). After the approval, patients were invited to participate in the study. All patients enrolled gave their informed consent for the collection and processing of data for scientific purposes.

## Author Contributions

The corresponding author of this manuscript is PO. PO, SD, CV, and AC participated in the study design. All authors have directly participated in data collection during surveys. MR and NJP carried out statistical analyses. NJP discussed results of analyses with PO and CV. NJP wrote the final version of the manuscript. All authors gave important suggestions for the final version of the content of the article, read its final version and approved it.

## Conflict of Interest Statement

The authors declare that the research was conducted in the absence of any commercial or financial relationships that could be construed as a potential conflict of interest.
